# Simulated Forest Immersion Therapy: Methods Development

**DOI:** 10.3390/ijerph19095373

**Published:** 2022-04-28

**Authors:** Amy Miner Ross, Reo Jane Francesca Jones

**Affiliations:** School of Nursing, Oregon Health and Science University (OHSU), 3455 S. W. US Veterans Hospital Road, Portland, OR 97239, USA; jonesre@ohsu.edu

**Keywords:** shinrin-yoku, virtual reality, phytoncides, exposure science methods, NK cells, Visual Analog Scale, DASS, BASDAI

## Abstract

Shinrin-yoku, forest bathing, may provide relief from chronic and breakthrough pain in patients with axial spondyloarthritis and improve immune function through increasing NK cell numbers and activity and their downstream effectors, perforin and granulysin, after chemo- or radiation therapy in breast and prostate cancer patients. The aim of this paper is to describe the study protocol for a simulated forest immersion therapy using virtual reality and atomized phytoncides, volatile organic compounds found in forested areas designed to effect positive change for these two patient populations. The setting, including the room set up and samples with inclusion/exclusion specific to this type of intervention, is outlined. Measures and calibration procedures pertinent to determining the feasibility of simulated forest immersion therapy are presented and include: ambient and surface room temperatures and relative humidity in real time, ambient ultrafine particulate matter, ambient droplet measurement that coincides with volatile organic compounds, specific phytoncides, and virtual reality and atomization of phytoncide set up. Particular lessons learned while training and setting up the equipment are presented. Simulated forest immersion therapy is possible with attention to detail during this early phase when development of methods, equipment testing, and feasibility in deploying the intervention become operational. The expected outcome of the development of the methods for this study is the creation of a standardized approach to simulating forest therapy in a controlled laboratory space.

## 1. Introduction

Shinrin-yoku, roughly translated as forest bathing, is the traditional Japanese practice of immersing oneself in nature by mindfully using our senses, such as sight and smell [1,2]. Shinrin-yoku practices range from sitting quietly and enjoying the forest to walking or hiking through forested areas [3]. The key to shinrin-yoku is connecting with the atmosphere of the forest, taking in forest aerosols, volatile organic compounds known phytoncides [4]. Phytoncides are inhaled airborne particles that trees naturally emit during different stages of development [5,6,7,8,9].

In 1982, the Japanese Ministry of Agriculture, Forestry and Fisheries began researching the health benefits of shinrin-yoku [2]. There are several therapeutic effects of shinrin-yoku in the context of depression, including improving immune system function, such as decreased pro-inflammatory cytokine activity and increased in anti-inflammatory cells, decreased depressive symptoms, stress, and anxiety, improved mental relaxation and attentional focus, and increased feelings of awe, gratitude, and selflessness [4,9,10,11,12,13,14,15,16]. The benefit of forested greenspaces for human psychology and physiology is a reduction in stress [14,17], which in turn positively impacts mood [18] and further reduces inflammation [19]. In the context of normal male subjects, after both shinrin-yoku forest immersion and hotel/sleep-based phytoncide humidification with α-pinene, β-pinene, and d-limonene, the following immune system improvements were found: natural killer T-cell numbers and activity increased, as did perforin and granulysin [6,20,21].

In 2017, a research agenda for nature contact and health was published with several research domains, including the mechanistic biomedical studies domain (1.0) for future research development [17]. We developed a simulated forest immersion therapy (SFIT) that includes both forest aerosols and virtual reality based on this research agenda. We are interested in psychological and physiological variables that suit our differing populations of interest (i.e., axial spondyloarthritis patients with chronic or breakthrough pain or breast and prostate cancer patients who have undergone chemo- or radiation therapy and have NK cell depletion), and these align with the mechanistic biomedical domain [17]. Specifically, our research objectives are to elucidate 1.1: to what extent stress reduction mediates observed health benefits of nature contact; 1.1b: which natural elements are most associated with stress reduction; 1.2: to what extent improved immune function mediates observed health benefits of nature contact; 1.2b which natural elements are most associated with improved immune function; and 1.2c which markers of immune function are the most useful for studying this effect [17]. If there are health effects of forest immersion, then could the same health benefits be achieved with simulated forest immersion as a way of providing therapy to patients not able to exercise or move in outdoor forests or greenspaces due to debility or frailty from chronic illness (i.e., chronic and breakthrough pain) or acute illness (i.e., recovery of immune system function after chemo- or radiation therapy in cancer)?

### 1.1. Significance

An estimated 1,806,590 new cases of cancer will be diagnosed in the United States each year, of which 281,550 will be breast cancer and an estimated 248,530 will be prostate cancer [22]. Additionally, up to 1% of the population of the United States, an estimated 2.7 million people, may have axial spondyloarthritis [23], and 50% of them suffer from chronic widespread pain [24,25] and breakthrough pain after standard treatment in approximately 60% of people [26]. Using complementary interventions to improve outcomes in patients who are seriously ill is paramount to extending healthcare to vulnerable populations. We hope to accelerate the translation of findings for healthy individuals for the implementation of a novel minimally invasive immune therapy for cancer patients with solid tumors where NK cells are depleted, both in number and activity [27,28], and to determine clinically meaningful protocols for the management of pain, and comorbid symptoms of pain, in patients with axial spondyloarthritis with chronic and breakthrough pain.

Forest bathing, virtual reality (VR), the use of phytoncides, and their separate or combined effects constitute a new research avenue. It is intriguing to explore the effects of nature-based interventions on chronic and breakthrough pain in patients with axial spondyloarthritis, as well as the effects on the immune system, and how we might harness them to benefit acutely ill patients who are immunocompromised.

### 1.2. Background

#### 1.2.1. Psychological Pathways of Interest

Contemporary theories, such as Kaplan’s Attention Restoration Theory [29,30], Ulrich’s Stress Reduction Theory [31,32,33], and Kellert and Wilson’s Biophilia Hypothesis [34,35] provide a conceptual framework for the practice of shinrin-yoku and engaging with nature in various forms of nature therapy. Shinrin-yoku researchers Song, Ikei, and Miyazaki (2016) developed a conceptual framework based on an extensive review that describes how the restorative effects of nature increase physiologic immune system recovery from stress as well as physiologic relaxation [36].

Kaplan and Kaplan hypothesized that exposure to natural settings through the five senses has a direct effect on parasympathetic nervous system activation, thus leading to states of greater awareness achieved through relaxation [29]. Ulrich’s Stress Reduction Theory [31] was developed from observational studies wherein patients in hospitals with patient room windows facing nature-laden scenery (e.g., trees, green foliage) experienced marked improvement in health and recovery with shortened hospital stays compared to patients in rooms with an urban view [31,32]. Wilson’s Biophilia Hypothesis [35], suggests that humans have a developmental affinity for natural surroundings, and being immersed in nature is therefore innately appealing. This research suggests that a disconnect from nature has adverse health impacts [34], and therefore finding effective means for individuals to access nature is crucial [37,38].

#### 1.2.2. Pain

Pain reduction is an emerging field of study in greenspace interventions [39], particularly the relationships between pain, stress, and the burden of chronic illness [40,41,42,43,44]. To date, several studies describe the pain-reducing effects of viewing nature (e.g., simulated greenspaces) in clinical settings for acute and chronic pain populations [37,41,45,46,47,48,49].

Simulated nature and greenspace exposure has been applied in clinical settings for the treatment of acute [41,46,47] and chronic pain [37,44,50]. Virtual reality (VR)-based therapies for pain reduction are not new, and several theories for how and why VR-based therapies improve pain outcomes center on the element of “distraction”, such that the virtual viewing experiencing distracts an individual from feeling their pain [51,52,53,54]. This is largely based on the Gate Control Theory proposed by Melzack and Wall [55], which suggests that the attention paid to the pain experience, as well as the emotion tied to the experience of pain, which includes past emotional memories, play a role in pain interpretation; therefore, directing attention away from the experience of pain may reduce the sensation of pain [47,53,56,57,58,59].

Research on VR-based greenspaces or nature exposure for pain reduction describes VR as a tool for delivering nature, and that nature is the crucial element within the interventional design [47]. In a repeated-measures design, 50 patients attending chemotherapy sessions were evaluated for pain and stress during intravenous port access. While findings were insignificant after one nature-based VR session, participants reported feeling relaxed, peaceful, and distracted by positive thoughts [47]. Potential benefits of virtual nature directly link to the theories describing the health effects of shinrin-yoku, including improved relaxation, restoration, and alertness, improved functioning of the immune system, and reduced exposure to air pollution and urbanicity [37]. Exposure to greenspaces can induce relaxation via psychoendocrine pathways, including the function of the hypothalamic–pituitary–adrenal (HPA) axis and resulting cortisol secretion [60,61]. Further, exposure to greenspaces, which include greenery in the form of foliage, trees, and vistas, such as with shinrin-yoku, improves health outcomes whether the exposure involves “live” nature or virtual nature [62,63].

#### 1.2.3. Biological Pathways of Interest

Immune suppression is a major issue for adults with a cancer diagnosis receiving chemo- and/or radiation therapy. In particular, NK cell suppression in this population is problematic as NK cells are the major immune cell type surveilling foreign or infectious antigens and eliminating them [64]. Thus, implementation of a novel minimally invasive immune therapy in cancer patients with solid tumors where NK cells are depleted, both in number and activity, is crucial [27,28]. Patients with solid tumors that have activated NK cells within the tumor have longer overall survival [65,66]. Blood levels of NK cells are essential to the movement of NK cells into tumor tissue.

Research shows positive effects of forest bathing on NK cell numbers and activity [67] (NK CD3^−^/CD56^+^/ and NK CD3^−^/CD56^+^/CD69^+^, respectively) and on expressed proteins, such as perforin and granulysin [6,20,68,69]. NK cells use pattern recognition molecules (epitope) on the surface of transformed or stressed cells to accelerate detection and elimination of problematic cells. Perforin and granulysin are key to enabling the natural killing mechanism of the NK cell [70]. Perforin is a downstream effector related to the number and activity of the NK cells [64]. Perforin creates a pore in the target cell once the target cell’s epitope is recognized [64]. The pore allows granulysin to enter the cell and effect apoptosis of the intracellular structures; the cell lyses and dies [5,64]. Perforin and granulysin are needed to maintain normal immune surveillance and reduction in infection, specifically in immunocompromised cancer patients [27,28].

Two proof-of-principle studies were conducted in middle-to-older-aged healthy men. These two studies, a 3-day forest experience (immersion experience) and a 3-night hotel experience, measured or used humidified-forest-derived volatile organic compounds, known as phytoncides, respectively. Of the phytoncides tested, humidified α- and β-pinene and limonene in combination produced an increased number of NK cells and elevated activity [6,20].

### 1.3. Purpose

If forest immersion can provide immune system benefits in healthy men (i.e., improved NK cell numbers and activity, increased perforin and granulysin), can dispersal of three phytoncides (α- and β-pinene and limonene in combination) paired with a greenspace virtual reality provide the same positive effects on NK cells in patients with solid tumor cancer who have completed cancer therapy? Additionally, can humidified limonene paired with virtual reality reduce pain and psychological stress in patients with axial spondyloarthritis? Our purpose is to deploy a standardized study protocol for simulated forest immersion intervention in cancer patients with NK cell depletion and patients with axial spondyloarthritis with chronic and breakthrough pain to test its feasibility and rigor. The simulated forest immersion intervention will provide greenspace/forest experience through three of the five senses. Virtual reality will provide visual and auditory stimuli. Humidified aromatic forest oils will provide olfactory stimuli. Virtual reality and atomized forest oils may be used in combination or alone [71]. Two distinct studies will use this standardized protocol for (1) cancer patients and (2) patients with axial spondyloarthritis. The purpose of this paper is to outline the development of the study protocol for the intervention and the control conditions of the clinical lab setting.

## 2. Methods/Approach

### 2.1. Research Design

We will use a two-arm study design with concurrent controls selected from the breast and prostate cancer clinics and from the arthritis clinic with two measurement time periods to test the proposed simulated forest exposure intervention. In the SFIT study with patients with axial spondyloarthritis who have chronic or breakthrough pain, the two-time points were before and immediately after the intervention. In the SFIT study with patients with either breast or prostate cancer, the two time periods were before and on Day 3 after the intervention.

### 2.2. Study Sample

The study sample for study #1 will be recruited from cancer patients with solid tumors (HR + HER2- breast, or prostate cancer) who have completed cancer therapy (hormone therapy excepted) as this population may benefit the most from increases in NK cell number and activity, perforin, and granulysin to prevent infection as patients become relatively immunocompromised after chemo- or radiation therapy. For study #2, the study sample will be recruited from axial spondyloarthritis patients who have chronic or breakthrough pain, as virtual reality has been shown to reduce pain, and d-limonene administration in animals has shown pain reduction. Since the two studies are set in the future and are pilot studies, we expect for study #1 to recruit and enroll 25 participants and for study #2 to recruit and enroll 25 participants. For study #1, the participant number is limited by budget and the cost of pre-clinical and clinical laboratory tests. For study #2, the participant number is limited by budget and the cost of paper-based tools. Concurrent controls for both studies will be identified by the clinicians in either the cancer clinics or the arthritis clinic. Concurrent controls will meet the same study inclusion and exclusion criteria as those who are enrolled in the study interventions. We expect to have 4 of each of the assigned genders in the control groups. The control groups will be randomized to receive neither SFIT intervention, VR, or atomized phytoncides. They will be exposed to atomized water dispersal for 1 h, the same length of time as the study participants who will receive the SFIT interventions. All the same data will be collected for both study #1 and study #2 on these control participants. At the end of the studies, should an effect of the SFIT intervention be noted, the control participants will have the opportunity to complete the same intervention. Clinicians will lead the recruitment of these patients, followed by a phone screening for inclusion and exclusion conducted by the principal investigator and research associate.

#### 2.2.1. Inclusion and Exclusion for SFIT—General Considerations

Since we will be using atomized phytoncides as well as virtual reality, either in combination or separately, several exclusion criteria apply, as seen in Table 1.

##### Inclusion and Exclusion for SFIT for Breast and Prostate Patients

Participants will be included if they are willing and able to provide informed consent, are of either biological sex, older than 18 years of age, and have completed cancer therapy for HR + HER2 breast cancer or prostate cancer, Stage I–III, with no evidence of metastasis. Participants will be excluded if they have a history of autoimmune disease, are on immune modulating therapies (endocrine therapy allowed), have had surgery or an invasive procedure in the past two months, and recent infection in the past two weeks (these are known confounding variables in immune system measures of interest).

##### Inclusion and Exclusion for SFIT for Axial Spondyloarthritis Patients

Participants diagnosed with axial spondyloarthritis (axSpA) will be included if they are willing and able to provide informed consent and are at least 18 years of age or older, and they may be any sex or gender. Additional inclusion criteria are: a score of 4 or higher on the 10-point Bath Ankylosing Spondylosis Disease Activity Index (BASDAI) (a standard criterion for suboptimal control of symptoms and disease [78] with a correlation between patient-reported BASDAI scores and measurable disease) [79], and a rheumatologist overseeing their care. Participants will be excluded if they are in an active phase of treatment with biologic cytokine inhibitors (which may confound the effects of the intervention on outcomes measures) [80]. Use of commonly prescribed painkillers is acceptable, and we will control for their use in the analysis.

### 2.3. Setting

The SFIT Lab is located in our Integrated Bio-Behavioral Lab space within our school. The lab room, where the instrumentation for the SFIT is set up, is 20 feet × 15 feet with a 12-foot ceiling. The room has temperature control, so a consistent temperature between deployments of SFIT with participants can be maintained, as well as lighting control, so the lights can be dimmed when patients are using VR.

### 2.4. Intervention—Procedure

**Figure 1:**
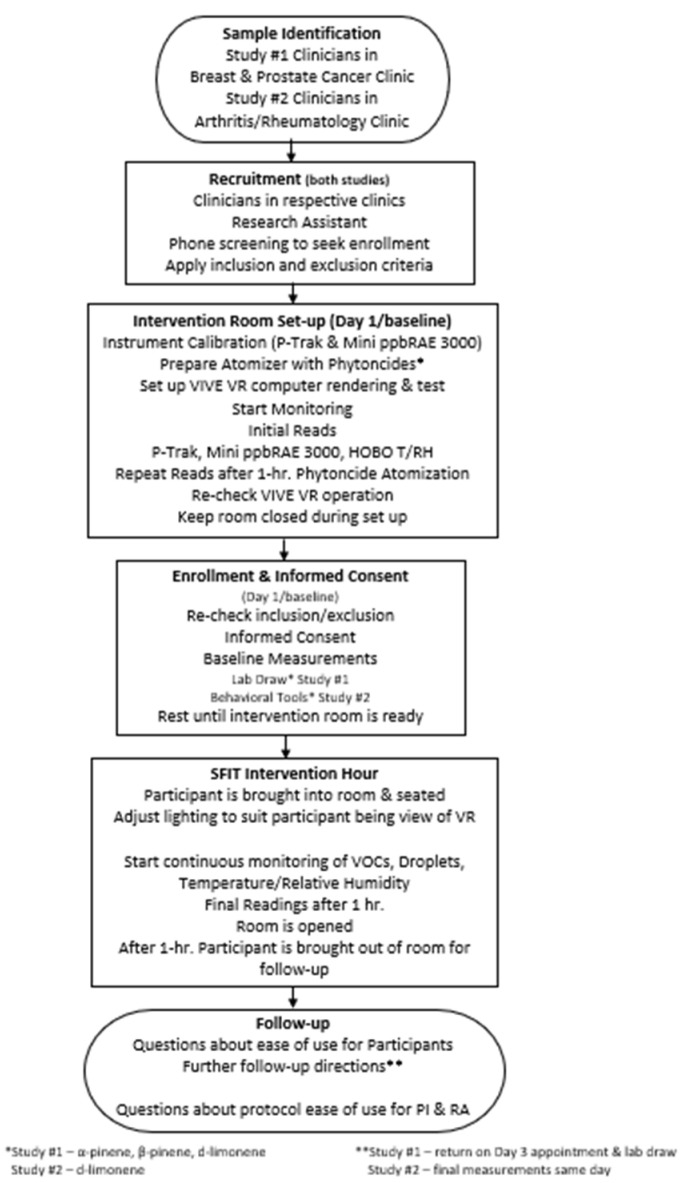
SFIT process/procedure.

Our two studies are novel as no one to date has used simulated forest immersion in patients with acute or chronic morbid conditions. The principal investigator (PI) and research associate (RA) will implement SFIT in a separate room from the room used to cross-check inclusion and exclusion, obtain informed consent, collect baseline measures, and allow the participant to rest. Prior to the arrival of the participant, the SFIT intervention space will be prepared. Preparation of the intervention space includes calibration of the instruments that will measure volatile organic compound particles (phytoncides are volatile organic compounds) and droplets, room temperature and humidity, and room surface temperatures, followed by measurement of the ambient particles and droplets and room temperature and humidity prior to the implementation of the atomized phytoncides [71,81]. Phytoncides α-pinene, β-pinene, and d-limonene (Floraplex Terpenes, Ypsilanti, MI, USA) will be prepared for atomization with a commercially available atomizer (Asakuki 500 mL Premium Atomizer, Tronhon Co., Ltd., Chongqing, China) that can emit phytoncides for up to 3 h. Once the dose expected (0.80 ppm) reaches the dose published by Li [6], the participant will be brought into the intervention room. To date, the Li study, which was conducted with healthy men, has been the only study to record phytoncide dose in a controlled setting. We will use this concentration as the target dose for our humidified phytoncide set up. Both at the beginning and at the end of 1 h of exposure to the simulated forest immersion therapy intervention, ambient phytoncide in the contained space will be measured indirectly by measuring both the increase in ambient air particle mass and number, as well as by measuring the change in total volatile organic compounds (VOCs). Total particle numbers will be measured with a continuous ultrafine particle counter (P-Trak 8525, TSI, Shoreview, MN, USA), and the total VOCs will be measured with the portable handheld monitor (Mini ppbRAE 3000, Honeywell International INC.; San Jose, CA, USA). The P-Trak and Mini ppbRAE 3000 will measure the increase in particle numbers and water droplets (aerosol), respectively, which will serve as a surrogate of relative exposure to phytoncide. Continuous monitoring of room temperature and relative humidity will be measured by the HOBO MX2301 Temperature/RH Data Logger (ONSET, Bourne, MA, USA). Room surface temperature will be measured by ADC Adtemp Mini 432 Non-Contact Infrared Thermometer (American Diagnostic Corporation, Hauppauge, NY, USA). VR will be provided by VIVE Pro Eye, HTC (high-tech computer) Corporation, (New Taipei City, Taiwan) with digital rendering of forested greenspace. Once 1 h of SFIT concludes, the participant will be removed to the preparation area and given instructions related to reporting unanticipated problems, adverse events, and serious adverse events, and an appointment to return for follow up on Day 3. See Figure 1, which illustrates the SFIT process/procedure.

#### 2.4.1. Intervention—Equipment and Calibration

All instruments will be placed horizontally on a table in the center of the room against one wall with sampling ports directed towards the participant for optimal measurement and dispersal of phytoncides.

##### P-Trak 8525, TSI, Shoreview, MN, USA

The P-Trak is a continuous ultrafine-particle (UFP) counter. The P-Trak has the capability of measuring particles as small as 100 nm. UFPs are currently studied to find associations with specific health effects [82]. The P-Trak will be zero-calibrated for each use using a charcoal filter. Both during the calibration and survey mode, research-grade isopropyl alcohol will be used in a small alcohol cartridge chamber in the instrument. The P-Trak has a data log that updates every minute and has a minimum and maximum range that is noted when the instrument is in survey mode. The data log will be downloaded onto a computer using software specific for the P-Trak [83]. All software will be accessed from the P-Trak website [81].

##### Mini ppbRAE 3000, Honeywell International Inc., San Jose, CA, USA

The ppbRAE 3000 measures volatile organic compounds (VOCs) related to the phytoncides that we are atomizing. The ppbRAE 3000 will be zero-calibrated using a charcoal zero filter and Isobutylene Air Balanced span gas [84]. For two-point calibration, isobutylene at 10 ppm and 100 ppm will be used [85]. During two-point calibration, we will use a set 0.5 LPM regulator. This regulator can handle up to 500 psi, which is the psi of the span gas cylinders and also within the toleration limit of the ppbRAE 3000 regarding pressure and flow. It is important that the gas cylinder connection is CGA 600 and corresponds to the connection on the regulator, meaning that the threading has to be compatible between the cylinder and the regulator. If 500psi is exceeded during calibration or survey mode, the diaphragm within the RAE 3000 may be damaged, leading to inaccurate data collection. In survey mode, data will be updated every 60 s and a data log will be created. The data log will be downloaded using software specific to the ppbRAE 3000 and found online [82].

##### HOBO MX2301 Temperature/RH Data Logger, ONSET, Bourne, MA, USA

The HOBO temperature and relative humidity (HOBO T/RH) data logger, which is suitable for both indoor and outdoor application, is a small portable unit that uses an application (HOBOConnect) loaded onto a mobile device. The app will use a Bluetooth connection to the HOBO T/RH that is easily configurable and logs temperature and relative humidity in real time that you will view on our mobile device, in this case an iPhone. The HOBO T/RH will be placed within a 30 m line of sight towards the participant. Due to the size of the room within which we will set up the intervention, we will use one HOBO T/RH. Data download will be accomplished when the iPhone (mobile device) is within 100 m of the HOBO T/RH unit. Data updates every 2 min with an accuracy of ±0.2 °C and ±3.5% RH [86]. Data software will be downloaded online [84].

##### Measurement of Ambient Room Conditions

Room temperature and humidity may alter the overall measurement of particle number and droplets. Measuring all four of these ambient conditions will allow for consistency in the experimental condition between participants. The room has a set temperature of 70 °F, and since the room is located against a foundation wall, humidity may vary; therefore, it is important to monitor both of these ambient conditions and use cutoff criteria based on average temperature and humidity of the controlled lab setting. We will also use a non-contact infrared surface thermometer to measure radiant heat of room surfaces that may add to the perceived comfort of the SFIT intervention room [81]. The ADC Adtemp Mini 432 Non-Contact Infrared Thermometer (American Diagnostic Corporation, Hauppauge, NY, USA) with a range of 59–77 °F will be used for this purpose. The ambient room air temperature, humidity, and surface temperature date will be collected as a mean prior to, during, and at the end of the intervention.

##### Asakuki 500 mL Premium Atomizer, Tronhon Co., Ltd., Chongqing, China

The atomizer holds 500 mL of liquid that can be atomized over 3 h. Of that 500 mL, a portion will be reduced that coincides with the amount of phytoncide that will be added. We expect that we will add 30 mL per phytoncide to the atomizer to achieve the detectable published amount [6,21]. Mist will be created by an ultrasonic plate within the atomizer, and the mist will be adjusted for a weak mist or a strong mist. Choice of weak or strong mist will be adjusted to fit the published detectable amount of phytoncide. Mist time can be regulated to maintain 60, 120, and 180 min of operation. We will use a 60 min mist time with the participants of study #1 and study #2. A fan within the atomizer will disperse the mist into the room. Room temperature and humidity will be monitored continuously as low temperature and high humidity may condense the mist into water droplets [87], which is to be avoided to allow for accurate dose calculations.

##### Phytoncides, Floraplex Terpenes, Ypsilanti, MI, USA

α-pinene, β-pinene, and d-limonene are the phytoncides (forest oils) of interest for SFIT. Interestingly, all three in combination have been tested in normal males in both forest immersion and hotel/sleep contexts and have shown effective elevations in NK cell numbers and activity as well as increased expression of perforin and granulysin [5,6,20,21]. However, these three phytoncides have not been tested in the SFIT context with breast and prostate cancer patients. D-limonene alone has been tested in the context of pain and shown to be effective in an animal model when not paired with VR [88]. All three phytoncides are available as purified isolates in containers of 4, 8, or 32 ounces. The phytoncides will be added to the Asakuki atomizer with an easy calculation of ounces to ml, and that amount will be subtracted from the 500 mL total container in the atomizer so as to maintain a standardized addition of phytoncides:water ratio. Measurement of this mixed mist by ultrafine particulate and VOC survey will be the method of determining the dose of the phytoncide.

##### VIVE Pro Eye, HTC (High-Tech Computer) Corporation, New Taipei City, Taiwan

The VIVE Pro Eye is capable of delivering digitally rendered greenspace visual recordings from forested or park-like greenspaces. The headset has sensors that coordinate the virtual greenspace with the participants’ visual gaze (native eye tracking) to move the surroundings of the digital greenspace through interaction with the base stations using motion sensors mounted on tripods in front of the participant [89,90]. Set up will include downloading the VIVE and SteamVR software onto a computer specifically dedicated for the VIVE system (e.g., using Windows 10 operating system). Tracking will be performed on the computer software and saved for later review during data entry [91]. The computer will be located behind the chair in which the participant will be sitting. Sounds will be adjusted for those that are a part of the virtual greenspace; ambient sounds in the room will be muted. Although software comes with the purchase of the VIVE system, training videos can be found online and will be completed before use [87,88,89].

### 2.5. Baseline Fidelity Measures—Linkages to Equipment

Perception of air quality prior to introduction of phytoncides into the room’s air will be evaluated repeatedly prior to each intervention day using two healthy individuals of both assigned sexes each time to assess the air quality of the room at the set temperature of 70 °F while monitoring the relative humidity. We will use the facial exposure method as described by Fang, Clausen, and Fanger [92]. In order to ensure fidelity of the measures of phytoncide dose prior to, during, and at the end of the intervention period, the equipment mentioned above will be zero and span-calibrated before introduction of phytoncides into the room’s air. Recording temperature and humidity using the HOBO T/RH before and during the intervention will ensure that the reliability of the survey data from the P-Trak and the ppbRAE 3000 has not been affected by changes in temperature and humidity.

#### Feasibility and Reliability of Intervention Stability

We expect to determine the ease of use of VR and phytoncide atomization, the drop off of phytoncide over the intervention period, and engagement in VR leading to a standardization of the procedure and protocol. Data will be collected from the participant, and the research associate and principal investigator will use field notes and will include challenges and facilitators related to the delivery method of VR and phytoncides and the ability of the participant to engage in VR for the duration of the study intervention. Atomized phytoncides prior to and after 1 h of dose delivery will be used to measure by the P-trak and the Mini ppbRAE3000 to determine dose drop off during the intervention delivery. Quantitative data related to dose drop off will be analyzed by *t*-test with an α level of 0.05. We will collect deviations from the standardized procedures, including the deployment of VR and atomized phytoncides. We will monitor the timing, preservation, and delivery of specimens to specialized labs on the academic healthcare campus in order to track the ability to maintain the expected optimized rigor in testing immune cells.

### 2.6. Data Collection

Since the participants will be recruited from the breast and prostate cancer clinics and the rheumatology clinic, the medical history that appears in the EPIC electronic medical record will be available for review prior to enrollment per IRB approval (OHSU IRB#00023183) and cross-checked with the participants after we have obtained informed consent on the day of the SFIT intervention. This will serve as the start of the data collection process for determining inclusion/exclusion of participants (see Section 2.2.1, Table 1; Inclusion and Exclusion for SFIT for Breast and Prostate Patients and Inclusion and Exclusion for SFIT for Axial Spondyloarthritis Patients) and baseline data collection (see Section 2.6.1, Section 2.7, and its subsections, and Section 2.8 and its subsections). Case report forms will be used to capture participant baseline and data collected at all pertinent time points per protocol and will serve as a hard-copy record of data, which we will enter into a research electronic data capture system, as required by our university.

#### 2.6.1. Baseline

Demographic data collection will occur prior to the start of both SFIT intervention studies and will be specific to each population of interest [93]. The behavioral/psychological measures, biological measures, and feasibility measures will be collected prior to placement of the participant in the SFIT intervention room.

#### 2.6.2. Follow Up

Follow-up data collection will be within the 3–4-day period after the SFIT intervention and in study #1 participants with breast or prostate cancer and will include blood specimens for CBC with differential counts of leukocytes, NK cell phenotyping and plasma for perforin and granulysin ELISAs [6]. We will survey the participants on events that might affect immune response. Every effort will be made to minimize the amount of blood drawn. Data collection will occur immediately after the intervention for study #2 participants with AxSpA and include the same measures as baseline as well as the intervention fidelity measures. Follow up will also include collection of adverse events, serious adverse events, and unanticipated problems, per IRB protocol for intervention studies.

### 2.7. Measures—Behavioral/Psychological

To measure the impact of SFIT on patients with chronic or breakthrough pain due to axial spondyloarthritis (axSpA), scales which interpret the direct effect on symptoms of pain, psychological distress, and physical functionality specific to axSpA will be used. These include the Visual Analog Scale for pain [94,95], the Depression, Anxiety, and Stress Scale [96,97], and the Bath Ankylosing Spondylitis Disease Activity Scale [98].

#### 2.7.1. Demographics

Demographic characteristics of participants will include clinically relevant ethnographic details specific to assigned sex (male or female), race, and ethnicity. Diagnostic information specific to axSpA, including date of diagnosis and onset of symptoms (date), chronicity of symptoms (in months), and non-biologic medication management (name of medication/last date of use), will be ascertained. Since culture, religion, and personal belief systems influence perception of pain, depression, stress, and functionality, we will include questions about these three pertinent individual characteristics in our baseline demographic data collection [99].

#### 2.7.2. Visual Analog Scale (VAS)

The VAS is a widely used self-reported tool measuring present-state perceived pain intensity [100]. Patients will be asked to indicate their perceived pain intensity along a 10 cm horizontal line (which can be on paper or computerized), and this rating will then be measured from the left edge up to the indicated marking to represent the level of pain intensity. The line represents a continuum between “no pain” and “worst pain”. The VAS is often used in clinical settings and is sensitive in determining the effect of comfort or pharmacological interventions [94]. The VAS has performed well on psychometric tests of validity (for example, η^2^ = 0.47; F = 0.44 [94,101]), and reliability (r_s,VAS_ = 0.52–0.89 [102]) for measuring pain clinically. VAS scores will be treated as ratio data [103].

#### 2.7.3. Depression, Anxiety, and Stress Scale (DASS)

The DASS comprises a set of three self-report scales, which are intended to measure clinically significant symptoms of emotional states of depression, anxiety, and stress [96,104]. Each of the three DASS scales (depression, anxiety, and stress) contains 14 items, divided into subscales of 2–5 items measuring the same construct, for a total of 42 items. The participants will be asked to complete the DASS prior to and immediately after the SFIT intervention. The DASS, which is intended to measure symptom severity of self-reported negative emotional states, including depression, anxiety, and stress, shows good psychometric validity and reliability (Cronbach’s *α* = 0.89; test–retest and split-half reliability scores are r_DASS_ = 0.99 and 0.96, respectively [96]) as a dimensional measurement of psychological distress associated with chronic conditions [104].

#### 2.7.4. Bath Ankylosing Spondylitis Disease Activity Index (BASDAI)

The BASDAI is commonly used to measure clinical symptoms of AS and axSpA, including fatigue, spinal pain, joint pain related to swelling, and enthesitis, or inflammation of the tendons and ligaments, as well as morning stiffness duration and severity [105]. It consists of 6 self-report questions, with each question scored from 1, representing “none” or no symptoms, to 10, representing “the worst”, with the score from the questions pertaining to morning stiffness and duration averaged such that 5 questions in total are scored. The participants will be asked to complete the BASDAI prior to and immediately after the SFIT intervention. The resulting score (from 0 to 50) is divided by 5 to give a final BASDAI score of 0–10, with scores of 4 or greater indicating significant disease [106]. The BASDAI has demonstrated extraordinary reliability at *p* < 0.001 [105]. In a test of validity of the BASDAI for AS patients, Cronbach’s *α* = 0.786 [98].

### 2.8. Measures—Biological/Immune System

We will use established pre-clinical and clinical measures to characterize the immune responses before and after the simulated forest exposure intervention.

#### 2.8.1. Demographics

Demographic characteristics of participants will include clinically relevant ethnographic details specific to age, assigned sex (male or female), race, and ethnicity, and history of smoking.

#### 2.8.2. CBC and Differential Cell Count

Complete blood count (CBC) and differential cell counts will be measured at baseline (prior to implementation of the simulated forest exposure intervention) and at Day 3. Correlation between this clinical measure and the data from flow cytometry and ELISA (outlined below) will be conducted to translate the pre-clinical findings into clinical use. A whole blood sample for a CBC with a differential cell count will be collected two times using a 4 mL EDTA tube, prior to the SFIT intervention and on Day 3 after the SFIT intervention. This measure will include WBC count and percentages of 100 cell counts and absolute counts for neutrophils, lymphocytes, and monocytes. The clinical core laboratory at OHSU complies with established inter- and intra-assay parameters as it is accredited by Clinical Laboratory Improvement Amendments.

#### 2.8.3. Flow Cytometry for NK Cell Number and Activity

Flow cytometry is used to monitor immune system changes tied to specific disease states, which makes it ideal for defining cellular responses of interest [107]. NK CD3^−^/CD56^+^ and NK CD3^−^/CD56^+^/CD69^+^, (i.e., NK number and activity, respectively) will be measured by flow cytometry immunophenotyping using freshly collected peripheral whole blood (approximately 4 mL). Cells will be prepared for flow cytometry using the standard fluorescence-activated cell sorting method. Data analysis will be performed by gating on live cells based on forward versus side scatter profiles, then on singlets using forward scatter area versus height, followed by cell-subset-specific gating [107].

#### 2.8.4. Perforin Expression

Perforin expression will be measured by an enzyme-linked immunosorbent assay (ELISA) and will be used to monitor downstream perforin secretion due to NK cell activity [27]. Perforin will be measured using plasma extracted from whole blood, which will be frozen at −80 °C and stored until needed for the assay. Optimized ELISA kits from ThermoFisher Scientific, Waltham, MA, USA, will be used per manufacturer instructions to detect perforin levels. The enzyme-dependent color change will be read out on a Multi-Mode Mircroplate Reader. Perforin concentration will be extrapolated from the standard curve [108].

#### 2.8.5. Granulysin Expression

Granulysin expression will be measured by an enzyme-linked immunosorbent assay (ELISA), which will be used to monitor downstream granulysin secretion due to NK cell activity [27]. Granulysin will be measured using plasma extracted from whole blood, which will be frozen at −80 °C and stored until needed for the assay. Optimized ELISA kits from AbCam, Cambridge, MA, USA, will be used per manufacturer instructions to detect granulysin levels. The enzyme-dependent color change will be read out on a Multi-Mode Mircroplate Reader. Granulysin concentration will be extrapolated from the standard curve. For both perforin and granulysin expression, we will need 4 mL of freshly collected peripheral whole blood [108].

### 2.9. Follow-Up Measures Day 3

In addition to collecting whole-blood specimens for CBC with differential, NK cell number and activity, perforin, and granulysin, we will note any unanticipated problems, adverse events and serious adverse events affecting the participants per IRB protocol for intervention studies. Unanticipated problems will be determined with the assistance of the clinicians and the study team as these are determined through a ranking procedure specified by our university’s Office of Human Research Protections. Adverse events will include subjective or objective symptoms occurring spontaneously, significant clinical lab abnormalities, a worsening of the participants condition from baseline, are recurrence or increase in signs and symptoms of original disease that occur after the SFIT intervention and are worsened or changed in quality. Serious adverse events will include death, life-threatening adverse event, new hospitalization or prolongation of current hospitalization, or a new significant incapacity or new substantial inability to complete activities of daily living.

## 3. Discussion

We have presented our lab set up for the SFIT intervention. The SFIT intervention as described may be used in a multi-arm design with a control group, using VR only, phytoncide atomization only, or both in combination. We expect to use whatever combination is proven to be most effective as a minimally invasive intervention for all of the following designs in a stepwise progression: longer intervention duration to optimize dose effect; intermittent, but repeated intervention to optimize dose effect drop off; and home use application to move the optimized intervention into practical use.

An intervention using VR and humidified phytoncides, α- and β-pinene, and limonene, in a simulated forest exposure intervention as a substitute for forest bathing in AXSpA patients with chronic or breakthrough pain and cancer patients with early-stage solid tumors (HR + HER2- breast cancer and prostate cancer) who have completed surgery or chemo- and/or radiation therapy (exclusive of hormone therapy) is possible in a lab setting. Moving SFIT to a home setting may be challenging, but not insurmountable.

### 3.1. Expected Outcomes

The expected outcome for the future two studies is the creation of a standardized protocol for deploying SFIT. This will include calibration and measurement set points, cutoff criteria and describing how to maintain a consistent dose of phytoncide and ease the use of VR for participants. We expect to uncover pertinent adverse events, severe adverse events, and unanticipated problems. For study #1, we expect that the combined use of our three phytoncides of interest and VR will improve NK cell numbers and activity and blood levels of perforin and granulysin in patients with breast or prostate cancer. For study #2, we expect that the use of d-limonene and VR will reduce pain, stress, and depression in patients with axial spondyloarthritis.

### 3.2. Lessons Learned

We have had challenges with procurement of supplies during the current development and delivery backlog produced by delays in shipping due to COVID-19 conditions in our current world state. Some of these were delivered relatively quickly, within one week; however, we procured span gas of the wrong concentration, and the next week all span gas was out of stock nationwide with a delay in the expected delivery of 1–2 months. Phlebotomy supplies have been challenging to obtain through our medical supply process within the university due to the increased usage of the supplies to care for COVID-19 patients. At times, our academic medical center announced requests for a reduction in the usage of various supplies for laboratory work. The lesson learned here is to start early after IRB approval and be aware of potential delays that might encumber grant funding previously awarded.

The cylinder containing the span gas connection and the fitting to the regulator must match. If the cylinder has a male threading and the regulator has female threading, these may match, but care needs to be taken in looking at the specification of the cylinder connection with respect to the regulator connection. In our case, we needed to have male threading on the replacement span gas with a connection that was a CGA 600 specification in order to match our existing regulator. The replacement price differential is 12:1 with the regulator being much more expensive than the span gas cylinder.

## 4. Conclusions

We have presented the theoretical conceptions and established the foundation for the move to the pragmatic operations of the SFIT intervention. SFIT is in its early stages as a potential therapy in the two populations of interest to us presented here. We expect further development in building this novel lab set up in the immediate foreseeable future as we work to move this therapy into the home setting under the control of patients needing this minimally invasive therapy. This is relevant to healthcare science because healthcare providers are responsible for optimizing patient healing and recovery, while reducing the harmful effects of therapies that deleteriously affect the patient’s ability to thrive with their chronic or temporarily morbid conditions.

## Figures and Tables

**Table 1:** General exclusion criteria related to intervention only.

Exclusion Criterion	Rationale
History of asthma [72]	Inhaled phytoncides may produce airway irritation, asthma exacerbation, or bronchoconstriction
Inability to detect common odors from commercial fragrances [73]	Inhaled phytoncides provide half of the intervention and smell of the forest
History of smoking within 15 min before the start of SFIT [74]	Smoking within 15 min before therapy will alter the ability of the participant to detect commercial fragrances or the aroma of the phytoncides
Allergy to pine or citrus aroma [74,75]	Inhaled phytoncide aromas are pine and citrus and may cause dermatitis
History of intractable seasickness [76]	VR may cause nausea/vomiting without relief after 5–10 min
History of seizures [77]	VR may heighten susceptibility to photosensitive seizure due to changing light in the forest video
Limitations of vision and hearing not corrected by eye lenses or hearing aids	VR requires good vision and hearing correction with eye lenses or hearing aids
Inability to complete study requisites	Intervention directions must be followed; specific to follow up measurements

## Data Availability

Not applicable.
